# In-Mold Sensors for Injection Molding: On the Way to Industry 4.0

**DOI:** 10.3390/s19163551

**Published:** 2019-08-15

**Authors:** Tatyana Ageyeva, Szabolcs Horváth, József Gábor Kovács

**Affiliations:** Department of Polymer Engineering, Faculty of Mechanical Engineering, Budapest University of Technology and Economics, Műegyetem rkp. 3, H-1111 Budapest, Hungary

**Keywords:** injection molding, in-mold sensors, process control, Industry 4.0

## Abstract

The recent trend in plastic production dictated by Industry 4.0 demands is to acquire a great deal of data for manufacturing process control. The most relevant data about the technological process itself come from the mold cavity where the plastic part is formed. Manufacturing process data in the mold cavity can be obtained with the help of sensors. Although many sensors are available nowadays, those appropriate for in-mold measurements have certain peculiarities. This study presents a comprehensive overview of in-mold process monitoring tools and methods for injection molding process control. It aims to survey the recent development of standard sensors used in the industry for the measurement of in-mold process parameters, as well as research attempts to develop unique solutions for solving certain research and industrial problems of injection molding process monitoring. This review covers the established process monitoring techniques—direct temperature and pressure measurement with standard sensors and with the newly developed sensors, as well as techniques for the measurement of indirect process parameters, such as viscosity, warpage or shrinkage.

## 1. Introduction

Industry 4.0 in the injection molding sector implies thorough process control. According to Karbasi and Reiser [1], full control of injection molding involves three levels: Machine parameters, in-mold parameters and part quality control. Today, a set of automation and digitalization technologies are developed to perform process control at each level. Among these technologies are on-machine and in-mold sensors, artificial intelligence methods, such as machine learning, big data, neural networks and many others. To satisfy the needs of Industry 4.0, a great deal of data for manufacturing process control is required. Although the in-line data coming from the machine is of high value for process monitoring and control, the most accurate processing data comes from the mold where plastic parts are formed. Therefore, in-mold process control is of vital importance. This review paper focuses on the acquisition of data by in-mold process technologies.

**Figure 1:**
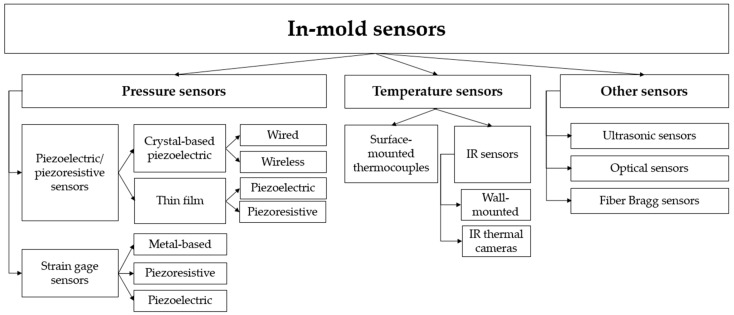
Classification of in-mold sensors.

In-mold process parameters are detected by sensors. Different kinds of sensors are available, which vary in measurement purposes and sensing methods. However, for in-mold process control, two classes of sensors are predominant—pressure and temperature sensors. A general classification of in-mold sensors is presented in Figure 1.

Injection molding is one of the most popular mass-production techniques for plastic production. Around one-third of the global production of plastics is processed by injection molding [2]. The measurement of process parameters in the cavity during injection molding poses several challenges. The first challenge is that melt-pressure normally exceeds 150 MPa. The second issue is that the sensing head is exposed to a corrosive and abrasive medium at high and fast-changing temperatures, frequently above 300 °C. The accumulation of a frozen layer during the cooling stage restricts measurement of the real melt pressure. Moreover, the sensors are usually embedded in a mold, which could cause output variations [3] as well as deviations of sensor dimensions and form due to the high temperature of the mold. Therefore, the proper selection of sensors for measurements in the cavity during the process is a challenging task.

This review aims to survey the current stage of development of in-mold sensors for injection molding and help researchers and engineers make the right choice when planning cavity measurements. The study covers the following sensors: Pressure sensors, including piezoelectric/piezoresistive and strain gauge sensors, temperature sensors, including surface-mounted thermocouples and infrared sensors as well as other in-mold sensors.

## 2. Pressure Sensors

The possible methods of sensing pressure [3] include: The mechanical deflection of a flexible member under a varying load; strain gauges, which measure the resistance of a folded wire deformed by pressure; sensors based on piezoresistive or piezoelectric effects; the variable capacitance of a diaphragm that deflects with pressure and a vibrating element that changes its resonance with pressure. Among the above-mentioned types, the most commonly used cavity pressure sensors are piezoelectric sensors, piezoresistive sensors and strain gauges. 

### 2.1. Piezoelectric and Piezoresistive Sensors

#### 2.1.1. Crystal-Based Piezoelectric Sensors

The performance of crystal-based piezoelectric sensors is based on the linear electromechanical interaction between the mechanical and the electrical state of crystalline materials. Under the applied load, the surface of certain crystals (usually quartz) becomes electrically charged proportionally to the load. The most widely used piezoelectric sensors are quartz sensors, as such sensors demonstrate excellent linearity over a wide amplitude of applied loads. Moreover, quartz pressure sensors are very rigid and even at maximum load, their deflection is only a few micrometers [4]. These advantages led to the widespread application of crystal-based piezoelectric pressure sensors.

##### Wired Piezoelectric Sensors

**Figure 2:**
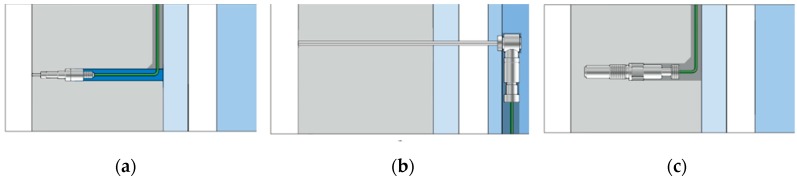
The principles of wired piezoelectric sensors: (**a**) Direct measurement: The melt pressure acts directly on the front of the pressure sensor; (**b**) indirect measurement: The ejector pin transfers the pressure to a force sensor; (**c**) contact-free measurement: Measuring pins capture the compression of the mold caused by the pressure [5].

Piezoelectric sensors can be used for direct, indirect and contact-free in-mold measurements. The direct measuring method implies that a sensor is in contact with the melt in the cavity and measures the pressure directly and without pressure loss. Most pressure sensors can be matched to the cavity surface, thus marks on the part surface can be prevented. The indirect measuring method is recommended when there is not enough space in the mold for a direct-measuring sensor. Contact-free cavity pressure measurements are required in cases when the appearance of marks on the surface of the part are strictly prohibited (class A surfaces, optical components, light conductors, etc.). Contact-free measurement is achieved with measuring pins [5]. The schematic representation of the principles of direct, indirect and non-contact measurement is presented in Figure 2.

Piezoelectric pressure sensors are available in different dimensions (1–15 mm sensor head diameter) and can operate in temperatures from −40 °C up to 400 °C and measure pressures up to 2500 bar. Piezoelectric pressure sensors are produced among others by Kistler, Priamus, RJG, Dytran, FOS and Baumer.

Wired piezoelectric pressure sensors are well developed and commercially available in numerous variations. These sensors are widely used for industrial process control as well as for different research purposes. In-mold pressure measurement can be used for simple cavity pressure and part quality monitoring [6,7,8,9,10], investigating the polymer melt flow and its rheological behavior [8,11,12], studying the effect of injection molding processing parameters on the pressure of the polymer inside the mold cavity [13], as well as the examination of various switchover methods [14]. 

##### Wireless Piezoelectric Sensors

A concept of wireless pressure sensors was developed by Kazmer and coworkers [15,16,17,18,19,20,21,22,23,24,25,26,27,28]. Their main motivation was to eliminate the expensive installation of wires through the mold to connect the sensor. There are two main concepts behind wireless pressure sensors: The use of ultrasound, which is able to propagate through the steel medium as an information carrier, and the extraction of energy for power supply through the conversion of mechanical energy from the manufacturing process into electrical energy. Kazmer and coworkers developed the design of the wireless self-energized cavity pressure sensor [15], which consisted of three components: The energy converter, the threshold modulator and the signal transmitter. The energy converter contains a multi-layer piezoceramic element, which generated an electrical charge proportional to the applied pressure. For the transmission of the obtained electrical voltage signal, they used sampling, quantization and encoding.

The authors also developed an analytical and a numerical model for the energy extraction process [16], designed and prototyped a multi-layered ultrasonic transmitter [17] as well as a threshold modulator [19]. Moreover, they modified the pressure sensor by introducing a temperature-sensitive oscillator module between the threshold modulator and the ultrasonic transmitter, thus enabling simultaneous melt temperature measurement by the same sensor [18]. Kazmer et al. [20] presented the experimental results of the dual-parameter wireless sensor application developed for pressure and temperature measurements when injection molding a 40 mm thick part. The obtained results demonstrated a pressure measurement resolution of 122 kPa and a temperature sensitivity of 4.8 kHz/°C, which is comparable to commercial temperature sensors [21]. The authors compared the pressure and temperature profiles obtained with the dual-parameter sensor they developed and commercial wired sensors [22,23]. The average error for pressure and temperature sensing was measured as below 4% and 5%, respectively. 

**Figure 3:**
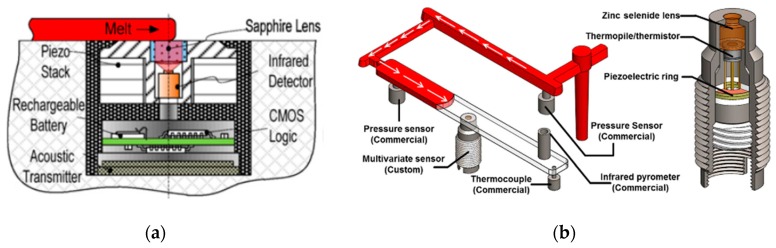
Multivariate sensor (MVS): (**a**) A schematic representation of a sensor (reproduced with the permission of Elsevier [23]); (**b**) MVS implemented on instrumented flex bar mold (reproduced with the permission of Springer Nature [25]).

Kazmer and Gao presented a multivariate sensor (MVS) with an IR detector for melt temperature sensing [24]. Later, the MVS was improved to measure additional injection molding parameters: Melt velocity, melt viscosity and mold temperature [25,28]. Melt pressure and temperature were obtained through the incorporation of a piezoceramic element and infrared photodetector, respectively, within the sensor head (Figure 3a). As a continuation of the work done in [28], the authors used the MVS they developed for quality control during the production of a flex bar [25,26]. To verify the MVS results, they flash mounted two commercial pressure sensors together with an IR pyrometer and a thermocouple into the mold (Figure 3b). They found that the MVS was able to measure pressure and temperature with an accuracy comparable to commercial sensors.

#### 2.1.2. Thin-Film Sensors

Another well-known mature piezoelectric technology is the integration of piezoelectric material in the form of thin-film in micro-electromechanical systems (MEMS) or resonators. Thin film technology allows the integration of a sensor system in machinery with a minimum change. The direct contact of the thin film sensor (TFS) with the workpiece ensures very precise process monitoring. Another advantage of TFS for process control is that several small sensors can be installed at different locations, thus monitoring the pressure at numerous points of the cavity. The chemical composition and purity of the TFS material determines its characteristics to a great extent, which allows for the tuning of the sensor’s pressure operating windows.

##### Thin-Film Piezoelectric Sensors

The most common piezoelectric materials for MEMS applications are ZnO and lead zirconate titanate (PZT). Both materials are rather difficult to process and integrate as a thin film with a silicon wafer. However, there has been some progress in the fabrication of PZT pressure sensors and their implementation for cavity pressure measurement. Luo and Chen [29] presented a fabrication process of PZT micro pressure sensor for in-mold cavity application. Thin-film sensors are manufactured by depositing PZT on the surface of a steel wafer at 300 °C by radio frequency sputtering, and the PZT is further annealed at 650 °C. Rapid thermal annealing ensures better ferroelectric properties (i.e., piezoelectric constant), as well as a better perovskite crystal structure of the PZT film, but at the same time, it can cause film cracks. Luo and Tsai [30] developed an integrated PZT pressure sensor array and a thin-film resistance temperature detector for the online monitoring of injection molding. The direct deposition method was used to integrate the PZT pressure array sensors and a k-type thermocouple on a silicon or steel substrate. The experimental results demonstrated the capabilities of pressure and temperature sensor arrays to monitor the related parameters of the core and cavity for the automatic adjustment of parameters and quality control of the product during injection molding. 

**Figure 4:**
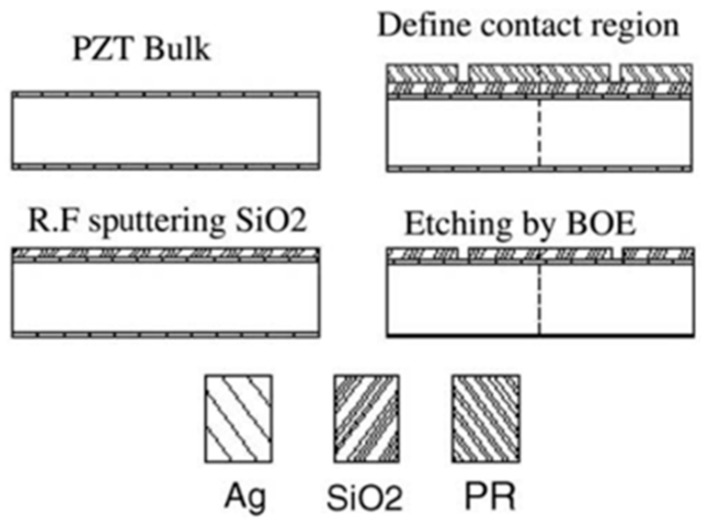
Procedure for making the lead zirconate titanate (PZT) sensor unit (reproduced with the permission of Elsevier [31]).

Another concept of PZT TFS was proposed by Huang and Cheng [31]. In order to prevent damage caused by high temperature, high pressure and corrosion in the mold cavity, bulk PZT was selected as the sensor unit instead of thin-film PZT. The concept of this sensor unit is schematically represented in Figure 4. The upper and bottom electrodes of the sensor unit were insulated to prevent electrical leakage [20]. They designed a measuring system to measure the output, which included qualitative analysis and quantitative analysis. The results were compared with the results of a Kistler sensor. It was proved that the new measuring system was very precise. The response times of the pressure sensor they developed and of the Kistler sensor were all less than 0.5 s.

Coates et al. [32] fabricated TFS by dispersing fine bismuth titanate (BIT) powder in a PZT solution directly in the mold insert with the following thermal treatment. The resulting BIT/PZT TFS was able to operate at temperatures up to 400 °C.

The relatively new piezoelectric material for thin-film sensors is aluminum nitride (AlN). Well-crystallized AlN exhibits a perfect c-axis orientation and consequently intense piezoelectric activity. Hence, it is predicted that AlN thin films will also be used for sensor applications [33,34]. However, high residual stresses often observed in polycrystalline AlN thin films make integration difficult. Dubois et al. [35] clearly showed that both the substrate and the deposition process significantly influence the growth and the properties of aluminum nitride thin films. They observed that the piezoelectric coefficient is very much influenced by the electrode material and the processing conditions. 

##### Thin-Film Piezoresistive Sensors

**Figure 5:**
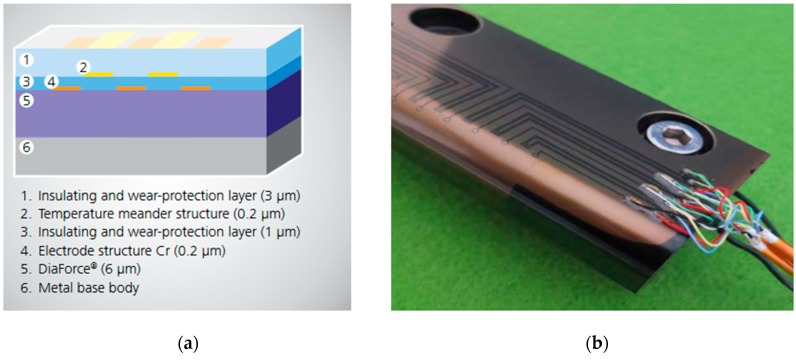
Multifunctional thin-film sensor system for cavity pressure measurements developed by Fraunhofer IST: (**a**) Functional layers of the sensor: 1—an insulation and wear-protection layer (material: SiCON®, d~1 μ); 2—a temperature meander structure (material: Chrome, d~0.2 μm); 3—an insulation and wear-protection layer (material: SiCON®, d~1 μ); 4—a lithographic structured metal layer (material: Chrome, d~0.2 μm); 5—a piezoresistive sensor layer (material: DiaForce®, d~6 μ); 6—a metal-based body; (**b**) general view of the thin film piezoresistive sensor [4].

The thin-film piezoresistive sensor is a newly developed type of pressure sensor. The Fraunhofer Institute for Thin Film and Surface Technology carried out important research on a novel type of sensor system, integrated into the mold cavity. They developed a piezoresistive TFS system (Figure 5a) based on an amorphous carbon layer (DiaForce^®^) [4,36]. The diamond-like coatings are optimized for an effective variation of the electrical resistivity depending on the applied mechanical forces. The complete system, including chromium electrode structures and an insulating wear-resistant coating, has a thickness of only 9 μm. The system is thermally stable up to 200 °C. Figure 5b shows a general view of the sensor system.

A comprehensive research project conducted in the framework of the project SensoFut [4] aimed to develop high temperature-high pressure sensor coating. Finally, a full sensor array, including a temperature sensor, was successfully fixed on injection molded inserts using all the developed sensor and insulating coatings. The obtained results demonstrated that the developed piezoresistive sensor was sensitive enough to indicate injection pressure in a robust and repetitive way. The sensor indicated the exact position of the thermoplastic melt front and at the same time as the Kistler piezoelectric sensor. However, the signal was highly influenced by temperature and therefore it was necessary to take a simultaneous temperature measurement and to adjust the signal to extract only pressure. In sum, piezoresistive TFSs for in-mold cavity pressure measurement are at a relatively early stage of development, and their feasibility and applicability in an industrial environment is still being studied by the research community. 

### 2.2. Strain Gauge Sensors

A strain gauge sensor is basically a metallic foil (or other material) pattern mounted on a diaphragm that deforms when exposed to the pressure of the melt. The mechanical force is converted into a voltage proportional to the strain. Strain gauges demonstrate superior high-temperature performance and lower cost compared with traditional piezoelectric sensors. However, strain gauges demonstrate a higher response time (up to 3–5 ms) compared with piezoelectric sensors [37].

All strain gauges for cavity pressure measurement can be divided into direct and indirect strain gauges. In direct strain gauges, the pressure applied by the medium is forwarded to the measuring diaphragm via the separating diaphragm and the transmission medium (usually mercury) in the capillary. The operating principle of indirect strain gauges is similar to that of indirect piezoelectric sensors. Cavity pressure causes the ejector pin to force itself against the sensor, which is mounted behind the head of the pin in the ejector plate. Depending on the material of the patterned metal, strain gauges can be divided into piezoresistive and piezoelectric strain gauges.

#### 2.2.1. Metal-Based Strain Gauge Sensors

The most widely used strain gauge sensors are metallic sensors. They are commercially available from various companies (RGJ, Dynisco, cavity Eye, Gefran and others) in different sizes (from 12 mm). The operating temperatures for metallic strain gauges can reach up to 400 °C, and the measured pressure can be as high as 2000 bar.

#### 2.2.2. Piezoresistive Strain Gauge Sensors

The change in the electrical resistance of semiconductors under mechanical load is up to two orders of magnitude greater than in metals [38]. The primary advantage of piezoresistive strain gauges over metal ones is their higher sensitivity, opening up completely new applications. Consequently, piezoresistive strain gauges allow pressure measurements at significantly higher frequencies, faster rise times, lower signal levels and at a wider range of temperatures.

Kulite Semiconductor Products, Inc. describes two types of semiconductor-based sensors intended for both static and dynamic pressure measurements. The first type is the silicon strain gauge mounted on a diaphragm. An integrated resistor is formed by means of diffusion or implantation (*n-*type or *p*-type) on a silicon diaphragm, which serves as the mechanical structure. The diffused elements form a Wheatstone bridge, which becomes unbalanced upon pressure, producing a proportional voltage. However, such kinds of piezoresistive sensors have a relatively low operating temperature (up to 150 °C) [39]. Although still in use, this technology has been superseded by the integrated sensor design.

Later, Kulite Inc. developed and patented the ‘silicon on insulator’ (SOI) technology, a variant where the piezoresistive elements are molecularly bonded to a micromachined silicon diaphragm with an insulating layer of SiO_2_ in between. This development extended the maximum operating temperature from 150 °C up to 600 °C.

Another type of well-known piezoresistive pressure sensors widely used in different applications (aerospace, automotive, manufacturing processes, etc.) are produced by Kistler. They are reported to have pressure and temperature operating windows of 0 to 0.3 GPa and −50 °C to 500 °C, respectively. Kistler piezoresistive sensors utilize a silicon sensing element mounted within a high-integrity seal assembly which is fully isolated from the pressure medium by a welded stainless steel diaphragm. Such sensors are intended for static and dynamic pressure measurements. The pressure-sensing assembly features a unique sealing method [40], which enables the sensor to withstand multiple cycles without fatigue.

Semiconductor strain gauge pressure sensors are usually used for measuring pressure in harsh environments, such as engines and powertrains, air conditioning systems, fuel, water and oil pumps and ground and flight tests for aerospace. Such sensors are produced by Kistler, Kulite Semiconductor Products, Honeywell, Kalvico, Keller America and other companies.

#### 2.2.3. Piezoelectric Strain Sensors Mounted on the Surface of the Mold 

Another way to acquire a cavity pressure profile during injection molding is to measure mold surface strain with strain gauges. Guan and Huang [41] determined a quantitative relationship between cavity pressure and mold surface strain by means of regression analysis. The authors used a piezoelectric surface strain sensor (Kistler 9232A), mounted on the external surface of the fixed mold half to monitor mold strain in the horizontal direction during injection molding. The strain of the mold changed the distance between the two contact elements on the sensor. This change in distance was converted into a force acting on the piezoelectric materials, which, in turn, produced an electric charge proportional to this force. 

## 3. Temperature Sensors

In-mold melt temperature measurement is a challenging task, as a temperature sensor is necessarily embedded in the metal of the mold and a significant amount of heat is transferred from the sensor head to the surrounding metal. Consequently, temperature sensors may have a significant phase lag and steady-state error in the measurement of melt temperature. Two types of temperature sensors are common today: Thermocouples and infrared (IR) sensors. 

### 3.1. Surface-Mounted Thermocouples

Commercially available in-mold thermocouples are usually flush-mounted with the surface of the mold cavity to prevent them from being enclosed in the part and to meet aesthetic requirements. As thermocouples are metallic, heat conduction to the mold causes the sensor to effectively measure the mold surface temperature rather than the melt bulk temperature [28,42]. The effect of conductivity can be reduced by insulating the thermocouple head from the mold [43]. 

In-mold thermocouples have been primarily used to signal the arrival of the polymer melt, which has proved to be useful for controlling the switchover position [44,45]. Surface-mounted thermocouples typically provide little information about the melt, (melt temperature, viscosity, flow front velocity, etc.). However, some researchers made attempts to predict the melt state using data from thermocouples and applying numerical modeling [46].

Another option to measure melt temperature with thermocouples is to place them at different depths in the cavity [47,48,49]. However, due to aesthetic requirements, this methodology is not used in industry and is limited only to research purposes [50]. For example, Nicolazo et al. [51] used a tubular needle to guide an embedded micro-thermocouple into the mold cavity. The temperature probe consisted of a type K thermocouple with a diameter of 80 μm, which had a small heat capacity. The measurement results demonstrated good agreement with the results of numerical simulations. Liu and Su [52] designed and implemented two specific injection molds equipped with two types of temperature measuring devices. The first mold had three mesh-type devices consisting of sheathed K-type thermocouples mounted on metal wires. The mesh-type devices were located at different distances from the inlet point (i.e., 48 mm, 78 mm and 108 mm). The second mold had a tubular-type device consisting of tubular needles guiding embedded micro-thermocouples into the cavities. Nine orifices of 1.5 mm in diameter were bored through the mold. Through these, the temperature probes (sheathed K-type thermocouples with a diameter of 0.7 mm) were inserted into the mold cavity. The needles had a length of 100 mm and an internal and external diameter of 0.8 and 1.1 mm, respectively. Both layouts allowed the researchers to obtain the three-dimensional temperature fields in the depth of cavities throughout the whole injection molding cycle. It was found that the tubular-type device induced far less flow disturbance and provided accurate temperature profiles. 

Several companies commercially produce thermocouples for in-mold application. Among them, the most popular are Kistler and Priamus.

### 3.2. Infrared Temperature Sensors

**Figure 6:**
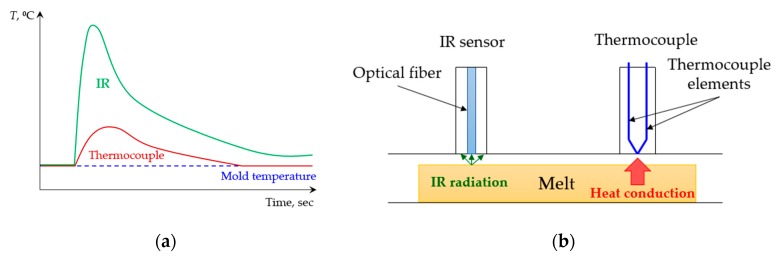
IR temperature sensors vs. thermocouples: (**a**) Comparison of the temperature measurement data obtained with thermocouples and with an IR sensor; (**b**) difference between contact and non-contact temperature measurements.

IR temperature sensors are free from the drawbacks of thermocouples. In these sensors, the radiant energy emitted by the melt is used to indicate the temperature inside the material. In contrast to thermocouples, IR sensors are not affected by heat conduction or heat convection and they also provide a very rapid response (Figure 6a). Moreover, non-contact sensing allows the measurement of the temperature of the product even when the resin contracts away from the sensor tip. Melt temperatures can be accurately measured throughout the filling, holding, cooling and mold opening phases (Figure 6b). However, other factors, such as the absorbance of the material complicates IR pyrometry. IR temperature measurements in plastics typically provide a weighted average of the temperature throughout the thickness of the material. The IR thermocouple measures radiation not only from the surface of the melt but also from deeper layers. The influence of absorbance was investigated in study [53]. 

#### 3.2.1. Wall-Mounted IR Probes

**Figure 7:**
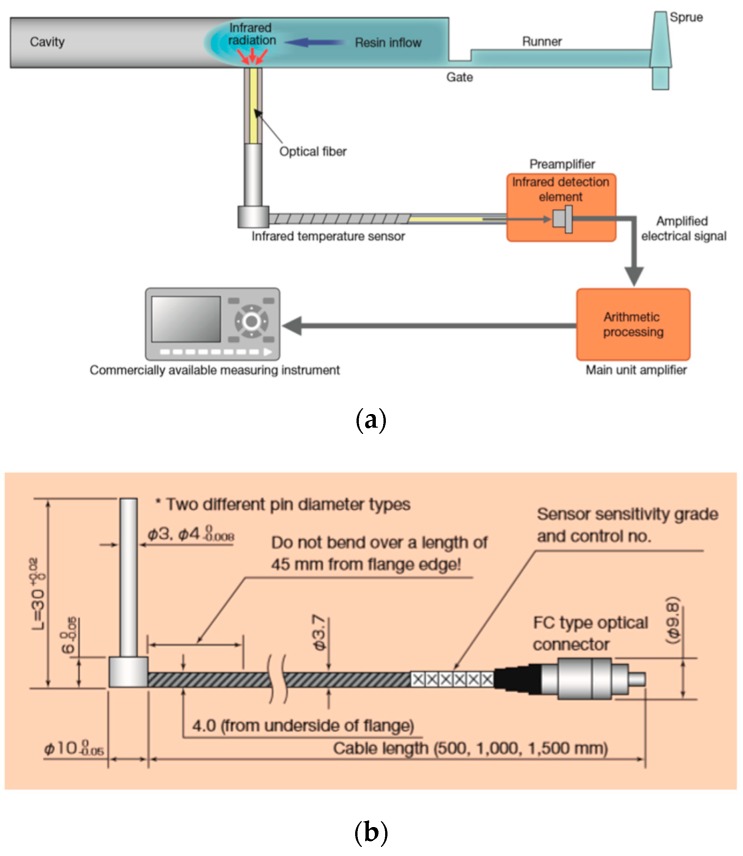

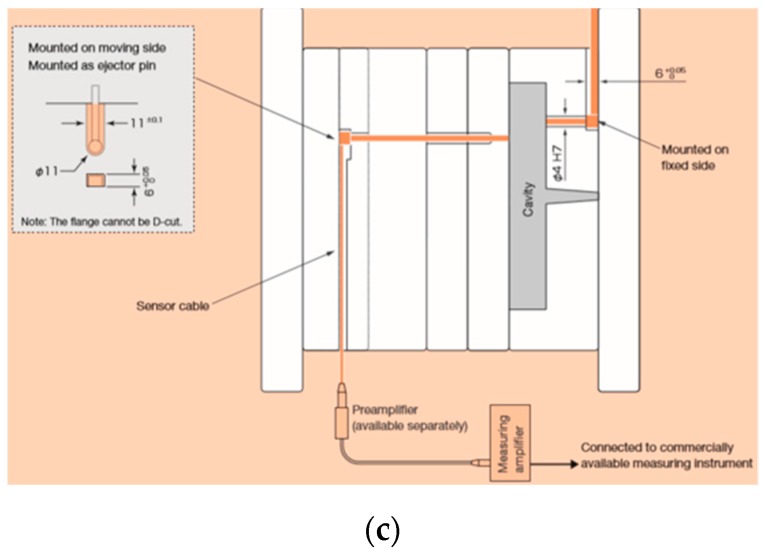
Working principle of an IR in-mold sensor: (**a**) Schematic representation of the installation of the whole system (e.g., sensor, amplifier); (**b**) schematic representation of an IR sensor (Futaba); (**c**) in-mold mounting example [56].

The working principle of a wall-mounted IR temperature sensor for in-mold measurement is presented in Figure 7a–c. The IR radiation emitted by the resin is conducted via an optical fiber to the preamplifier, where it is converted into an electrical signal. After conversion, it is processed by the amplifier and output as a temperature signal [54].

IR sensors provide a very fast response time (1–240 ms), which enables the detection of rapid temperature variations [55]. The main producers of IR thermocouples are Futaba Corporation (Oshiba, Japan), Calex Ltd. (Abingdon, UK) and Optris GmbH (Berlin, Germany). The head diameter of an IR sensor can be as small as 3 mm (Futaba). 

Despite the unquestionable advantages of IR sensors for in-mold temperature monitoring, they are not widely used in the industry. Today, the application of IR temperature sensors is mostly restricted to research purposes. 

Kazmer et al. [57] used an IR-sensor, which replaced an ejector pin to predict the dimensions of injection molded parts. The method they developed is based on feedback from stacked melt temperature and pressure sensors. 

Johnston et al. [50] used an Omega OS1562 sensor to validate the data obtained from thermocouples. Due to its rapid response time, the IR sensor detected the arrival of the melt as a nearly instantaneous change between its threshold value of 82 °C and the observed peak melt temperature. The bulk temperature measurements were not affected by radiant energy from the opposite mold wall (which was 3.2 mm away), as the coolant temperature was varied by 30 °C and a corresponding shift in the measured melt temperature was not observed. 

Pacher et al. [12] used three triple-combined FOS MTPS408 cavity sensors aligned along the flow path of the plate-shaped cavity to measure melt temperature. Each triple-combined sensor featured an IR probe, a type K thermocouple and a piezoelectric pressure detector. Such configuration enabled the local and transient recording of melt temperature, mold temperature and cavity pressure near the gate, the center of the part and the end of the part simultaneously. The temperature measurement results demonstrated a significant difference in polymer melt (measured by the IR pyrometers) and mold surface (measured by the thermocouples) temperatures just at the moment when the polymer melt front reached the sensor. This difference was illustrated by the peak of the temperature curve (see Figure 6) obtained by the IR sensor, and amounted to almost 100 °C in comparison with the temperature measured by the thermocouple.

Some researchers developed their own IR sensors for in-mold temperature measurements. For example, Bendada et al. [58,59,60] developed, manufactured and tested a hollow waveguide IR thermometer, which exhibited low transmission loss of thermal energy in the far and mid-infrared ranges, and had no end reflection. This hollow waveguide consisted of a silver film deposited on the inside of a smooth glass supporting tube, and a single fluorocarbon-polymer film which was transparent at the desired wavelength. The concept of the waveguide is based on the fact that although metal-tubing fibers exhibit high attenuation, a properly designed inner dielectric coating significantly reduces attenuation loss. The probe was installed in the moving side of the injection mold along with the detector head. The advantage of the developed hollow fiber is its high transmission in the far and mid-infrared bands, therefore it can be used for relatively low-temperature applications (e.g., room temperature), whereas conventional full-core optical fibers exhibit high transmission loss in those spectral regions, and are also very expensive and fragile. 

#### 3.2.2. IR Thermal Cameras

**Figure 8:**
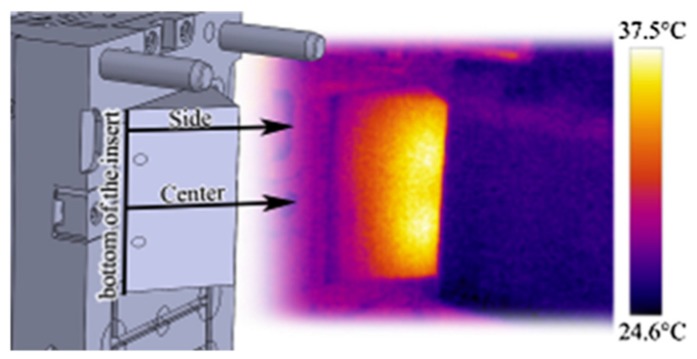
The thermal image of an injection molded part (reproduced with the permission of Elsevier [63]).

IR thermal cameras are often used to visualize cavities and hot injection molded parts (Figure 8). Such a camera can provide mold temperature maps, which show “hot spots” in plastic parts, thus indicating the distribution of unbalanced cooling zones. Additionally, thermal scans taken of plastic parts immediately after demolding provide important information on possible sources of part warpage [61,62]. 

Bula et al. [61] used a FLIR T620 IR camera to measure mold temperature during the injection molding process, and part temperature just after the mold opening. The data provided by the IR camera were also used for the verification of the results of numerical analysis [64]. 

## 4. Other In-Mold Sensors 

### 4.1. Ultrasonic In-Mold Monitoring

In-mold temperature and pressure sensors are widely used for process monitoring. However, some interesting and important process parameters and peculiarities cannot be identified with these types of sensors. A noninvasive and nondestructive ultrasonic technique can provide a great deal of information about the cavity during injection molding. It can shed light on such important research questions as local flow front arrival [65,66], the end of filling [32,67], the detachment of the injection molded part from the mold wall, gate freeze-off time [68,69], the development of the solid/liquid interface [70], the evolution of the morphology [71,72], melt homogeneity and temperature gradients [73], melt temperature [74] and the orientation of the polymer chain [75] during injection molding. Temperature and pressure profiles can also be deduced from the ultrasonic signal [76], and part thickness can be also measured with the acoustic sensor [77].

### 4.2. Optical Monitoring 

**Figure 9:**
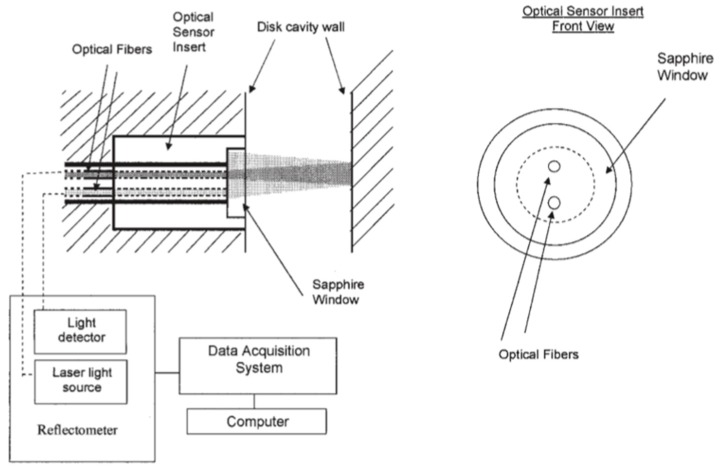
General scheme of the optical system for the monitoring of crystallization (reproduced with the permission of John Wiley and Sons [80]).

The cooling stage, at which the polymer crystallizes or solidifies, controls the injection molding cycle. Therefore, for process optimization, monitoring the crystallization of the polymer is essential [78]; this can be performed with optical measurements [79]. There have been several attempts to develop an optical sensor to monitor crystallization kinetics. Marinelli et al. [80] developed an optical sensor, which was installed on the cavity wall (Figure 9). Their measurements showed that the system was sensitive to changes in crystallization. They also observed that the temperature of the mold and injection temperature were the most influential variables of the crystallization kinetics of polybutylene terephthalate (PBT) and polytrimethylene terephthalate (PTT). Due to its slower crystallization kinetics, PTT was found to be more sensitive to changes of the injection molding variables than PBT. 

**Figure 10:**
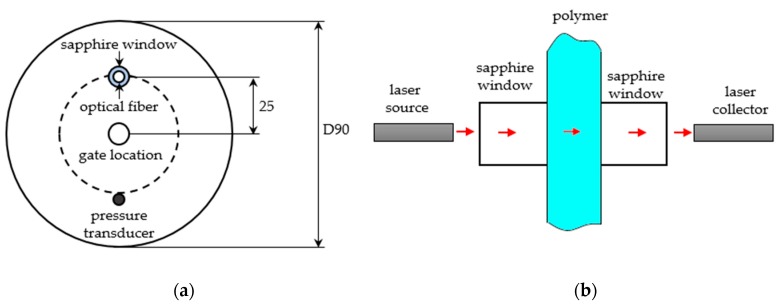
Optical sensor for in-mold process control: (**a**) Scheme of the mold cavity showing the optical device fibers and the position of the pressure transducer; (**b**) scheme of the path of the light of the optical device during injection molding (reproduced with the permission of John Wiley and Sons [75]).

Favaro et al. [78] developed and tested another kind of optical sensor. The optical fibers were inserted through a hole in the cavity, which was covered with a sapphire window, and was located 25 mm from the cavity center. At the opposite side, a Kistler 6152A pressure transducer was installed. The polymer was injected through an inlet point at the center of a cavity wall. At the opposite mold wall, an optical fiber cable was installed to collect the transmitted light (Figure 10). The optical sensor was sensitive to the differences in the polymer crystallization kinetics of nucleated and non-nucleated polypropylene (PP) resins and to the change of injection molding parameters on that kinetics. Later, the authors utilized the same sensor for monitoring the crystallization kinetics of intercalated polypropylene (PP)/clay nanocomposites during injection molding [81].

### 4.3. Fiber Bragg Gratings for Monitoring Injection Molding

Alberto et al. [82] used a set of multiplexed fiber Bragg gratings (FBGs) for two-dimensional monitoring of the thermal behavior of the mold. The results allowed them to identify the different phases of injection molding.

### 4.4. Melt Viscosity, Shrinkage and Warpage Measurements

#### 4.4.1. In-Mold Melt Viscosity Measurement

Changes in melt viscosity influence the pressure distribution in the mold cavity and have a strong impact on part quality, as viscosity fluctuations can cause differences in warpage or can even result in under-packed or over-packed cavities. That is why the monitoring of viscosity during the injection molding process is important. There are several methods for measuring melt velocity in the cavity. For example, melt viscosity can be measured with ultrasonic sensors [69], which detect the position of the melt front and melt velocity can be calculated. Based on the spatial propagation velocity of ultrasound in different materials, the value of the reflection and transmission coefficients presents the different interfaces between the mold and polymer or the mold and air. The Capacitive sensing method [83], based on the difference in dielectric property between polymer and air can also be used for measuring viscosity. This method involves an electrode mounted within the cavity, and the melt velocity is measured from the ramp rate of the sensor output with respect to time. However, short shots or air trapped in the cavity can result in an error of the measured capacitance. In injection molding, melt viscosity is in direct relation with pressure change. Another method to measure melt viscosity in the mold cavity is to use nozzle pressure measurements. Nozzle pressure is in direct relation with the shear stress of the melt, which is in a direct relationship with the flow rate. Therefore, viscosity can be determined as the ratio of nozzle pressure to injection rate, and if the flow rate is assumed to be constant, the only variable will be nozzle pressure [84]. Asadizanjani et al. presented an online viscosity monitoring method based on the measured velocity and pressure of the melt [85]. Melt velocity can be calculated with the use of the ramping rate of melt temperature and the time derivative of melt pressure.

#### 4.4.2. Shrinkage

Dimensional consistency is critical for the quality of injection molded parts is highly dependent on the morphology of the polymer, its thermal expansion and various processing parameters. A traditional way to estimate the dimensional shrinkage of the part is based on the pressure-volume-temperature (pvT) behavior of the polymer. Another method is a computer simulation. However, the pvT relationship for a particular material only helps decide the preliminary tolerances in part design and tool design. Available computer-aided engineering simulation software also provides only an estimation of the shrinkage based on pvT, the geometry of the part and the processing conditions. Therefore, none of the above-mentioned techniques can directly monitor and control shrinkage and part dimensions online [86]. 

Traditional hardware-based temperature and pressure transducers are widely used in the industry to predict shrinkage. However, a clear correlation has not been found yet between the measured progress of temperature and pressure, and product quality [6]. Speranza et al. [87] developed a procedure to calculate average solidification pressure, a parameter that is critical for the description of local shrinkage. They used a conventional pressure transducer to analyze the development of local pressure. The procedure is thus suitable for a master-curve approach in which some data for shrinkage versus average solidification pressure can be used as a reference. Kazmer et al. [57] developed a method based on the real-time feedback of melt temperatures and pressures, with the aim of predicting the change in the dimensions of parts during injection molding processes. The authors used the volumetric shrinkage values to estimate how much the molded part shrinks. These predicted shrinkage values can be used to reasonably estimate actual shrinkage levels. 

In-mold sensors can be very helpful for on-line shrinkage measurements. For example, strain gauges were adopted to follow shrinkage from the instant of the beginning of solidification [86,88,89], and optical fibers were proved to be able to measure thickness shrinkage [90,91]. 

##### Shrinkage Measurement by Strain Gauges

Titomanlio et al. [92] measured the shrinkage of an injection-molded polystyrene (PS) part with piezoelectric strain gauges (Kyowa KFRP-5-350-C1-1) placed inside the mold with the gauge axis aligned with the direction of the flow. During injection, the polymer solidifies on the strain gauge, which, afterwards, follows the shrinkage process inside the mold at that position. The measurements showed that shrinkage starts in the mold after about 7 s, and the samples suddenly shrink when the mold is opened, after about 25 s. The experimental results on PS showed that final shrinkage decreases if higher pressures are used; at the tip of the cavity, in-mold local shrinkage starts earlier and final shrinkage may be greater than near the gate; final shrinkage may decrease if geometrical constraints are placed inside the mold, as they prevent in-mold shrinkage. The authors also modeled injection molding with a software code developed at the University of Salerno (UNISA code), which takes into account crystallization kinetics [89]. They concluded that the shrinkage results were accurate if an absolute solidification crystallinity of ∼40% was chosen; such a result was consistent with the rheology change of the same material during crystallization. Using such a solidification crystallinity, the software was able to correctly predict shrinkage in both constrained and unconstrained cases and to accurately estimate the onset of shrinkage.

Panchal and Kazmer [86,88] developed a button cell type in-mold shrinkage sensor, and validated and compared it against traditional shrinkage prediction and estimation methods. The shrinkage sensor consisted of an elastic diaphragm instrumented with strain gauges connected in a full-bridge circuit. The sensor was placed beneath the movable pin, which was in the mold wall and in contact with the melt and remained in contact with the sensor diaphragm. The sensor diaphragm was deflected by the pressure of the melt acting on the pin in the mold cavity and was retracted back to its original position as the melt solidified and shrank away from the mold cavity wall. The authors analyzed the sensor signals acquired during each molding cycle as a function of packing pressure, melt temperature, cooling time and coolant temperature in a design of experiments, in order to validate the performance of the sensor. The regression results indicated that the shrinkage sensor outperformed cavity pressure transducers and other methods of predicting in-mold shrinkage. The authors stated that for polypropylene, the sensor they developed was able to measure shrinkage with an average accuracy of 0.01 mm for a molded part with a nominal thickness of 2.5 mm. The coefficient of determination between the thickness the sensor measured and the final thickness of the part was 0.921 for the in-mold shrinkage sensor. Other dimension prediction methods had lower correlation coefficients.

##### Measuring Shrinkage with Optical Sensors

**Figure 11:**
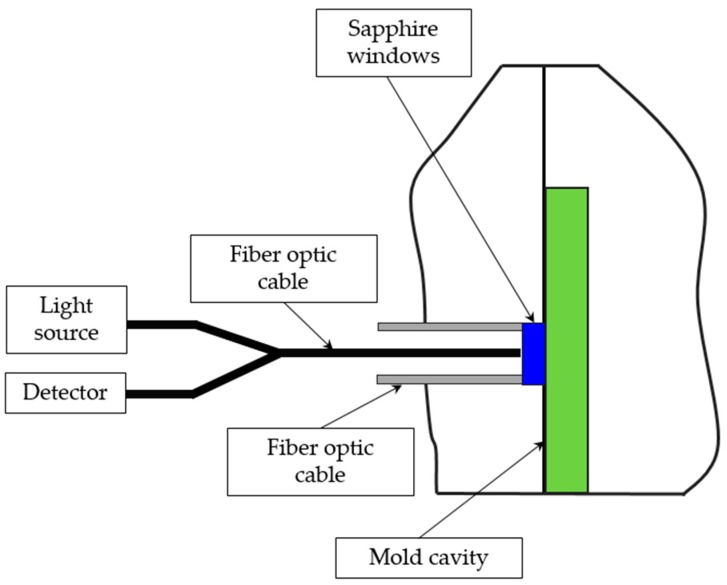
Schematic representation of an optical fiber sensor for in-mold shrinkage measurement (reproduced with the permission of John Wiley and Sons [85]).

Bur and Thomas [90,91] developed an optical fiber sensor embedded into the ejector pin channel that ends with the sapphire window (Figure 11). The optical cable consists of a bundle of nineteen 100 μm diameter fibers. Seven fibers transmit light from a laser and the rest of the fibers transmit reflected light back to a photodiode. During the injection molding process, the detected light is analyzed in order to show both the arrival of the flow front and the separation of the part from the mold wall upon shrinkage. 

#### 4.4.3. Warpage

Warpage is a dimensional distortion of a part after its removal from the mold. Warpage is a consequence of uneven stress distribution in the part caused by non-uniform mold temperatures. Warpage affects the surface quality and dimensional stability of the parts and therefore is of great concern. Several techniques have been developed for monitoring this parameter [93].

Huang and Tai [94] found that the parameter influencing warpage most was packing pressure, while the influence of the dimensions of the gate and filling time in thin shell injection molding is practically negligible. Zamani et al. [95] experimentally investigated the warpage of a thin and centrally-gated disk by means of melt-pressure marks of two different locations inside the mold cavity left by piezoelectric transducers. Ozcelik and Sonat [96] analyzed the warpage of PC/ABS parts in terms of melt temperature, mold temperature, packing pressure and packing time. Kong et al. [97] found out that warpage can be reduced significantly with a lower molding temperature and a smaller coefficient of thermal expansion. Mathivanan and Parthasarathy [98,99] designed a simple generic model to identify the most influential parameter and built a nonlinear mathematical model to predict sink mark depth. Selvaraj and Venkataramaiah [93] used the image processing tool of MATLAB for the measurement of warpage. 

## 5. Conclusion

Industry 4.0 requires a great deal of data for manufacturing process control. The most relevant data come from the mold cavity, where the part is formed. Therefore, it is essential to monitor process parameters in the mold cavity. Sensors for monitoring the two most important parameters of the injection molding process—temperature and pressure—have been developed through the decades, and nowadays different types of such sensors are available. The most popular pressure sensors are piezoelectric sensors and strain gauges, while the most popular temperature sensors are thermocouples. However, certain imperfections of the well-known measuring tools inspired researchers to develop new devices, based on ultrasonic, IR, thin-film and other technologies. The future of in-mold temperature measurement is most probably IR measuring devices, as they provide very fast response and, unlike thermocouples, measure the temperature of the melt directly. Piezoelectric crystal-based pressure sensors have demonstrated stable and reliable operation through the years and will most probably retain their leading position for in-mold pressure measurement in the future. However, thin-film piezoelectric and piezoresistive sensors seem to be a good alternative for conventional pressure sensors, as TFSs are very compact, easily installed and therefore save significant space in the mold.

Besides temperature and pressure, a number of important process parameters, such as viscosity, warpage, shrinkage and others are worth monitoring during injection molding. Therefore, many scientists and engineers work on developing methods and tools for monitoring these parameters. Some unique sensors, for example, an optical sensor for shrinkage monitoring, have been developed. However, such sensors are at a very early stage of development and need to go a long way to become a reliable industrial solution. 

## Abbreviations

ABS	acrylonitrile butadiene styrene;
AlN	aluminum nitride;
CAE	computer-aided engineering;
FBG	fiber Bragg grating;
IR	infrared;
MEMS	micro-electromechanical system;
MVS	multivariate sensor;
PBT	polybutylene terephthalate;
PC	polycarbonate;
PP	polypropylene;
PTT	polytrimethylene terephthalate;
pvT	pressure-volume-temperature;
PZT	lead zirconate titanate;
SOI	silicon on insulator;
TFS	thin-film sensor.
